# Advocating for Greater Inclusion of Marginalized and Forgotten Populations in COVID19 Vaccine Rollouts

**DOI:** 10.3389/ijph.2021.1604036

**Published:** 2021-10-11

**Authors:** Mirko Farina, Andrea Lavazza

**Affiliations:** ^1^ Faculty of Humanities and Social Sciences, Innopolis University, Innopolis, Russia; ^2^ Department of Neuroethics, Centro Universitario Internazionale, Arezzo, Italy

**Keywords:** vaccine distribution, ethical framework, marginalized populations, inclusion, equity

## Abstract

**Objectives:** Many countries recently approved a number of SARS-CoV-2 vaccines. There is therefore growing optimism around the world about their future availability and effectiveness. However, supplies are likely to be limited and restricted to certain categories of individuals, at least initially. Thus, governments have suggested prioritization schemes to allocate such limited supplies. The majority of such schemes are said to be developed to safeguard the weakest sections of society; that is, healthcare personnel and the elderly.

**Methods:** In this work, we analyse three case studies (incarcerated people; homeless people, asylum seekers and undocumented migrants). We propose a bioethical argument that frames the discussion by describing the salient facts about each of the three populations and then argue that these characteristics entail inclusion and prioritization in the queue for vaccination in their country of residence.

**Results:** Through an analysis informed by ethical considerations revolving around the concepts of fairness and equality, we try to raise awareness of these important issues among decision makers.

**Conclusion:** Our goal is to advocate for the development of more inclusive policies and frameworks in SARS-CoV-2 vaccine allocation and, in general, in all scenarios in which there is a shortage of optimal care and treatments.

## Introduction: Setting up the Problem

There are currently over 100 potential COVID-19 vaccines in some stage of testing and development around the world [1]. Across all these potential vaccines, the most effective ones, which have been approved for usage by a significant number of health agencies around the globe and are indeed being administered to vast swathes of the population are: Sputnik V, Pfizer-BioNtech, Moderna, Astrazeneca, and Sinovac Biotech.

These Covid-19 vaccines differ substantially with respect to the approach they use to prepare our immune system to spot and fight SARS-CoV-2, the virus that causes COVID-19, as well as with regard to the technology used for their development^1^.

For example, innovative RNA vaccines (such as the one developed by Moderna and Pfizer-BioNtech) use fragments of genetic material pre-prepared in the lab to code for a part of the virus; typically, in the case of SARS-CoV-2, the infamous spike protein. As the vaccine is administered to the patient, her body starts using instructions found in the RNA to build copies of this specific virus part. As the body recognises these parts, it starts mounting an immune response, which is effective to prevent the development of the symptoms related to COVID-19.

These RNA vaccines, although based on a technique which has never been used before in the history of mankind, appear to be very safe and secure [2, 3]. Nevertheless, they differ from more traditional vaccines, based on tried and trusted techniques, such as virus vector vaccines or inactivated vaccines.

Virus vector vaccines (such as Sputnik V and AstraZeneca) use a virus, typically weakened, attenuated, and unable of causing troubles to the host, to deliver -*via* injection-a virus antigen into the patient’s body. As the virus begin infecting cells, the host’s body starts expressing large amounts of antigens, which help it mounting a formidable immune response that protects the individual from catching Covid-19 [4].

Inactivated vaccines -also known as killed vaccines- (such as Sinovac Biotech) contain viruses which have been grown in culture, in this case SARS-CoV-2, and then killed (with heat, chemicals, or radiation) to destroy the disease producing capacity. So, these vaccines inoculate a virus into the host that cannot replicate but can still determine a strong immune response that protects the patient from getting the disease^2^.

The variety of techniques on which these vaccines are based as well as their different characteristics and protocols used for their administration shall ensure that COVID-19 vaccination is performed securely and effectively worldwide, for all members of society (including infants, the elderly, and immunosuppressed patients).

Nevertheless, as Covid-19 threatens more than seven billion humans inhabiting Earth, an effective vaccination campaign can only be accomplished (despite theoretical availability of vaccines) with top-notch logistics and excellent organisational skills [5, 6].

Alas, as we quickly found out, vaccine supplies are rather limited and their worldwide distribution (even among industrialised countries) is unequal [7, 8], being also guided by key geopolitical goals and interests [9]. In addition, certain types of vaccines (typically those based on RNA techniques) are quite expensive, being priced at about 30 dollars per shot, and require very low temperature (between −20 and −70 Celsius) for storage, which demands significant investments in technology to preserve their efficacy. This poses a mighty challenge for procurement to poorer, non-industrialised countries [10, 11].

In this context of shortage and utter uncertainty, many governments-advised by experts and public health agencies-have suggested prioritization schemes to allocate vaccines limited supplies in the most effective and fairest way; that is, to safeguard and protect the weakest sections of society (typically healthcare personnel and the elderly).

In this short contribution, owning it to lack of space, we cannot delve into the analysis of the important issue of vaccine unequal access on global scale [12]. Nevertheless, we focus on the related, but much more specific theme of prioritization schemes and protocols for vaccine distribution and administration in industrialised countries (typically EU and USA). In particular, we look (*Methods: Our three case studies* below) at the recommendations for priority vaccination developed by a number of western governments, institutions, and public health agencies (such as the UK government, the U.S. Centers for Disease Control and Prevention, and the WHO) as well as to some of the most influential advice given by leading researchers in the field [13, 14]. In analysing these guidelines, responses, and policies, which are laudable for their humanitarian purposes and aims, we nevertheless notice a troublesome absence -or at least a scarce attention-to developing criteria for vaccine allocation designed to ensure fairness of treatment and equality for some disenfranchised and disadvantaged groups [15, 16] belonging, especially, to the following three main categories: 1) incarcerated people; 2) homeless people, 3) asylum seekers and undocumented migrants. In this paper, we propose a bioethical argument that frames the discussion by describing the salient facts about each of the three populations and then argue that these characteristics entail inclusion and prioritization in the queue for vaccination in their country of residence, for several humanitarian and public health reasons.

Through a qualitative analysis informed by ethical and philosophical considerations revolving around the concepts of fairness and inclusiveness we then try (*Results: Fairness and Equality in COVID-19 Vaccine Distribution, Ethical and Philosophical Perspectives*) to raise awareness of these troublesome issues among decision makers and politicians alike. In doing so, we advocate and actively push (*Discussion: the Need for More Inclusive Policies in Vaccine Allocation in Cases of Shortage of Optimal Care and Treatments*) for the development of more inclusive ethical policies and frameworks in SARS-CoV-2 vaccines allocation, distribution, and administration and -in general-for all scenarios in which there is a shortage of optimal care and treatments.

## Methods: Our Three Case Studies

Because, as we have seen above, the supply of COVID-19 vaccine is quite limited, Public Health Agencies worldwide have made a number of recommendations to federal bodies and local governments about who exactly should be vaccinated first.

These agencies consist of independent bodies and panels of medical and public health experts and their recommendations are typically made in the interest of public health with a few basic goals in mind [17]. Some of these are: 1). decreasing the number of Covid-19 deaths or of people seriously affected by it; 2). reducing the spread of the disease 3); protecting health and social care staff and systems; 4). ensuring the functioning of society; 5). preserving the psychological integrity and well-being of individuals, and 6). reducing the extra burden that COVID-19 inflicted on people already facing other types of disparities (such as socio-cultural and economical).

In this section, we focus on the specific advice on vaccination priority developed by: (a). The U.S. Centers for Disease Control and Prevention^3^, and (b). The UK government^4^. As space is limited, below we provide a brief, yet substantial summary of the main points contained in the documents produced by such agencies/institutions. It is worth noting that the recommendations provided in these documents are significantly overlapping.

For example, all these guidelines abovementioned agree on first immunizing healthcare workers, who typically have very high risk of acquiring and transmitting Covid-19. All guidelines, given higher mortality rates, also agree on vaccinating -in the initial stages-the elderly (those aged above 75), specifically those residing in long-term care facilities. The categories that should be immunised in the second phase, according to these guidelines, include frontline essential workers (such as fire fighters, police officers, corrections officers, agricultural workers, manufacturing workers, grocery store workers, public transit workers), and those who work in the educational sector (teachers, support staff, and day-care workers) as well as people aged between 74 and 65, because they are at high risk of hospitalization.

As large quantities of vaccine become readily available the vaccination campaign, it is suggested, ought to expand in order to include more categories, thereby gradually immunising non-essential workers, people aged between 60 and 40 with underlying medical conditions or morbidities, and eventually the youngest sector of the population, which is normally less affected by Covid-19. Inspired by these general recommendations, several researchers designed a series of vaccine procedures or interventions, aimed at optimising immunization schedules.

For instance [13, 7: eabf1374] used “an age-stratified mathematical model to establish optimal vaccine allocation in relation to four metrics (deaths, symptomatic infections, and maximum non-ICU and ICU hospitalizations) under different scenarios.” The results of this study confirmed that vaccines ought to be first administered to high-risk groups (such as those indicated in the guidelines and recommendation we discussed above) and then allocated to high-transmission groups (e.g., younger individuals)^5^.

More recently, confirming and extending these results [14], developed a mathematical model to quantify the impact of COVID-19 vaccine prioritization strategies on cumulative incidence, mortality, and years of life lost. The results of this study show that after taking into account “country-specific age structure, age-contact structure, infection fatality rates, and seroprevalence, as well as the age-varying efficacy of a hypothetical vaccine” [14, p.918], those aged 60 or older ought to be immunised first in order to minimize deaths and hospitalizations.

We certainly do not intend to criticise these prioritization strategies and optimization schedules, which are based on sound empirical research and surely believe that they are excellent tools, which can help us immensely in regulating and minimising the effects of the current pandemic; however, from the guidelines and recommendations discussed above; we cannot help but noticing a troublesome absence, or at least a scarce attention, to some basic humanitarian and ethical criteria to ensure fairness of treatment and equality for disenfranchised and disadvantaged groups. We notice such an absence with respect to three main categories of people (incarcerated people; homeless people, asylum seekers and undocumented migrants).

In truth, in the “*Roadmap for prioritizing population groups for vaccines against Covid-19*” formulated in September 2020 by the WHO^6^, vaccination for at least two of these categories (detained people and homeless people) is envisaged. However, “social/employment groups unable to social distance (examples: detention facilities, dormitories, low-income persons in dense urban neighbourhoods, homeless people and those living in informal settlements or urban slums, certain occupations e.g., mining)” are to be vaccinated, according to WHO, towards the end of the second phase (“localized or limited transmission”, p.9) when vaccine availability become more widespread (ranging from 21–50%, p.10).

Yet, we know that people in correctional facilities are prone to acquire all sort of infectious diseases. In fact, epidemiological evidence attests that mass incarceration increases contagion rates for infectious diseases [19]. Further, inmates generally have poor health, including many co-morbidities that place the population in jeopardy should they contract the virus. It has also been shown [20] that incarcerated people are particularly vulnerable to Covid-19 because of a series of structural factors (such as overpopulation, confined spaces, and relatively poor sanitation) affecting the environment in which they dwell. For example, it is worth noting that an outbreak of COVID-19 at a prison can quickly overwhelm the local health system and put patients in the community at risk of not having appropriate healthcare available. However, if one chooses to prioritize this population -as we propose doing in this article-one can easily vaccinate all who consent since they are a captive population and this means that they can be reached as easily as residents of other congregant living arrangements (e.g., nursing homes).

Similarly, but perhaps less evidently, homeless people live in disadvantaged conditions, usually have suboptimal health statues (e.g., due -for instance-to alcoholism or substance abuse) with little or no access to proper medical care [21]. In addition, homeless people typically dwell in conditions that are conducive to virus transmission. In fact, many of those who experience homelessness regularly sleep in congregate settings (such as common shelters, ruined buildings, or encampments) and often experience lack of basic hygiene (such as showering). One could nevertheless object here that homeless people -contrary to incarcerated people-might be less affected by COVID-19 because many of them live outdoors in relative isolation. We recognise this as a potentially good objection. There is indeed an ongoing debate, especially in the U.S., about whether this is the case or whether instead homeless people simply die of Sars-Cov-2 at higher rates, uncounted and unnoted. To try to undermine this objection we can resort to official statistics. If we look at official statistics^7^, we see that homeless deaths rose sharply since March 2020 in many US States, just as the pandemic arrived. In particular, deaths tripled in San Francisco^8^, rose by 32% in Los Angeles^9^ and by 54% in Washington D.C^10^. In addition, it has been shown in important studies [22, 23] that the COVID-19 pandemic affected severely the life prospects as well as the physical and mental well-being of homeless people. These statistics suggest an urgency in vaccinating these people. More importantly, this evidence seems to demand that countries worldwide adopt a more inclusive stance and start devoting appropriate resources to the recruitment of these disadvantaged/disenfranchised populations (in many senses akin to racial and ethnic minorities) to vaccination. Nations should also use delivery systems that are likely to be trusted by these populations and are effective at administering it to them.

Finally, as a recent report by [24] has shown, asylum seekers and undocumented migrants, living in detention centres are at severe risk of developing Covid-19. Over the last year, dramatic COVID-19 outbreaks have been reported in refugee camps on Greek mainland, in detention centres located in Germany and Italy, and even in a hostel in Portugal (see also [25, 26]). In US detention centres there have been over 1,200 confirmed COVID-19 cases across 52 facilities, according to recent statistics [27]. Data about other types of migrants is scarce; however, it is reasonable to assume that -given their general lack of legal status-migrants cannot afford proper medical care, do not have access to state benefits (because they normally do not possess proof-of-income documents), hence are fully exposed to complications arising from exposure to Covid-19 [28].

A sceptical reader may object that even if undocumented immigrants and asylum seekers are usually held in detention centres (hence potentially affected more significantly by the COVID-19 pandemic); many of them also live in other settings with risk factors similar to those of the populations within which they live and work, e.g., low-wage essential workers. This is true; however, it is also worth noting that undocumented migrants fear being identified and expelled from the country they are in. For this reason, they do not access health services except in the event of a serious emergency or they resort to humanitarian organizations, who provide free healthcare without considering the origin and identity of their patients. Thus, under normal conditions, both undocumented migrants and asylum seekers experience difficulties in getting adequate health treatments and may suffer from diseases that are usually treated effectively in the rest of the population. In the case of the Covid-19 pandemic, the situation can be exacerbated. In fact, undocumented migrants and asylum seekers aren’t likely to go to vaccine distribution centers for the same fear that usually keeps them away from hospitals and medical centers. Furthermore, the vaccine won’t be available from non-governmental organizations. In addition, they will probably have more difficulties in accessing other contagion prevention tools (such as face masks, disinfectants, and gloves). For these reasons, it would be significantly easier for them to be exposed to the virus, to transmit it, with greater probability and greater incidence than other social groups.

In this sense, including undocumented migrants and asylum seekers among the categories to be protected with high priority is a measure that would favor both inclusivity and equality in treatment, while also helping society (by -for instance-avoiding the development of uncontrolled outbreaks of infections). This seems to be the idea underlying the decision taken by the UK Government in February 2021. According to such a decision, migrants will be eligible to receive vaccines irrespective of their current legal or working status in the country. However, this does not mean giving priority to migrants, in fact, “those living in Britain who entered the country illegally would be encouraged to register with their local doctor, so they could be vaccinated when their turn comes.”^11^

Surely, the three categories of individuals we described seem to be severely affected by the current pandemic, perhaps in no lesser way than the elderly and health care workers. Yet, policies and interventions developed by leading organisations and institutions (despite being informed by humanitarian concerns), have not given such categories adequate attention in vaccine allocation. We believe that these categories ought to receive more attention by public health agencies and experts. For these reasons, in pursue of fairness, equity of treatment, and inclusiveness in responses, we next call (*Results: Fairness and Equality in COVID-19 Vaccine Distribution, Ethical and Philosophical Perspectives*) for the development of a more inclusive ethical framework for vaccine allocation, distribution, and inoculation that is capable of taking into account the interests and needs of these disadvantaged/disenfranchised groups.

## Results: Fairness and Equality in COVID-19 Vaccine Distribution, Ethical and Philosophical Perspectives

There are indeed a number of crucial ethical values that are relevant to formulating fair and inclusive policies or frameworks for Covid-19 vaccine allocation [29]. Some of these values (such as adopting a principle of distributive justice in delivering Covid-19 vaccine to developing countries) are relevant to the issue of global access to vaccine allocation, on which we touched earlier on (*Introduction: Setting up the problem* above, see [30]). Some others (such as equality in treatment involves consideration of differences, such as gender, race, or religion) and apply, perhaps more specifically, to the way the vaccine is distributed and administered within a specific country, among different sectors of societies. Certainly, some of the values belonging to the latter category may well apply to the former category. However, our interest here is in building upon [12, 29, 31, 32] and further developing an ethical and philosophical framework revolving around the notions of fairness, inclusiveness, and equality [33], which is capable of safeguarding and better protecting those categories we specified above. We lay down some of the basic principles for such a framework below.

It seems unquestionable that ill-health is something everyone wants to avoid and there is therefore a widespread consensus that ill-health is bad. Thus, it seems reasonable to argue that there should be the chance for everyone to avoid ill-health. This does not mean that everyone can or should be in perfect health at every stage of their life, nor that there is an obligation for the state or citizens to guarantee perfect health for all. Yet, there seems to be a moral obligation to guarantee widespread remedies against ill-health, especially when it is particularly disabling for the individual.

However, it is also a fact that health conditions cannot always be easily rectified in every single moment in time. Therefore, the other elements that are preconditions for avoiding ill-health are also important and should be carefully analyzed. Among these elements, equality seems a fundamental one. In the case of Covid-19 one can ask what are the reasons in favor of equality. Following Scanlon [34], we can consider reasons for equality and inequality in a broader sense and in narrower sense.

The reasons belonging to the latter category, reasons in a broader sense, are “objecting to the difference between what some have and what others have” [34, p.1]. These kinds of reasons include the consequences of this basic difference. We can take into account effects that in themselves are not connected to equality in the strict sense. The classic example in this context is the empirical evidence that inequality has serious consequences on the health of those who are most disadvantaged [35]. These are reasons that see equality as an instrumental value, since the reasons for wanting to remedy ill-health are not of an egalitarian type in themselves.

But we can also desire the equality of citizens for reasons in the narrower sense. Reasons of this type are based on the idea that inequality is objectionable in itself and not for reasons related to its consequences. The principle for which we should combat inequality, for example in access to vaccines, is that this condition creates an “unacceptable degree of control over the lives of those who have less” [34, p. 2]. Another important principle, which we may want to consider in this context, is that of basic moral equality. According to this principle each person counts morally, has a moral status that entails certain rights (for instance, the right not to be interfered with in one’s life but also the right to receive help and support in some circumstances), which cannot be subordinated to individual differences related to race, gender, religion, geographical origin or preferences and lifestyles.

According to these two latter principles, all people should receive equal concern and the fact that inequalities are created in a country is problematic because it weakens the fairness of that country’s political institutions and people’s trust in those institutions. However, some might argue that the three groups we are considering here are not victims of inequality as discussed above; rather, they may believe that their condition is due to the behaviors in which they have engaged, which created the conditions for which they are now disadvantaged.

In this context, we can further distinguish between what [36] calls differential option luck and differential brute luck. The starting point is that egalitarianism has the purpose “to eliminate involuntary disadvantage” by which [ [37], p.916] “(stipulatively) mean (s) disadvantage for which the sufferer cannot be held responsible, since it does not appropriately reflect choices that he has made or would make.” According to [ [36] p. 293], “option luck is a matter of how deliberate and calculated gambles turn out—whether someone gains or loses through accepting an isolated risk he or she should have anticipated and might have declined”. Brute luck instead is “a matter of how risks fall out that are not in that sense deliberate gambles” [36, ibidem].

For example, if I lose the use of all my limbs due to a neurodegenerative disease I was born with, my brute luck is very bad. If I bet a million dollars on the lowest temperature of the year and I win, my option luck is pretty good [38]. In general, brute luck is linked to the unavoidable and includes events for which the agent has no ability to influence the probability as well as events for which the agent is unaware of her ability to influence the probability [36]. In spite of the meritocratic and individualistic perspective, based more on a prescriptive principle than on social research data, most of those who are among the homeless, inmates or undocumented migrants are people who have often had bad brute luck and not people who chose “the vices” and ended up in poverty or in prison or had to flee their country [39, 40].

However, one could argue that not all the members of the three categories we described above are in their condition due simply to “bad luck”. This objection could be raised for inmates. However, with respect to incarcerated people, one can argue that the punishment for their crimes does not include being more exposed than others to SARS-CoV-2. Given -as we have seen above-that prisons are places with a high risk of contagion; it is part of a principle of fairness to put inmates in conditions of equal risk to that of the rest of the population and, therefore, to vaccinate them with a high degree of priority.

## Discussion: The Need for More Inclusive Policies in Vaccine Allocation in Cases of Shortage of Optimal Care and Treatments

Vaccination plans that the majority of countries have drawn up are aimed at the safeguarding and protecting the most fragile sectors of society, while attempting to contain the spread of the infection. In addition, such plans are developed with ethical principles (such as the protection of the greatest number of lives) in mind, and in accordance to efficacy rules based on the best epidemiological knowledge and practice. However, in most of these policy plans, as well as in many real-life decisions taken during the Covid-19 pandemic, some disadvantaged groups (especially ethnic minorities) have been neglected. In this article we have shown that three specific groups (inmates, homeless people, asylum seekers and undocumented migrants) seem to be deserving more inclusive policies as well as greater attention and appreciation by governments and decision makers alike. These are groups that for different reasons, as described above, are both at greater risk of contagion and have a lesser chance of resorting to virus protection tools as well as to proper medical care.

These groups (especially homeless people and undocumented migrants/asylum seekers) are typically marginalized within society and may not know how to procure vaccination. For this reason, we believe that we ought to consider them as akin to racial and ethnic minorities. In other words, we shouldn’t just pursue (even though this is -we believe-a very laudable objective) the prioritization goal of achieving eligibility for vaccination at a level appropriate to the population’s risk level. We should do more; we should actively call for a deeper reform in public health policies; that is, we should advocate for more equity and inclusion at the level of national and supranational policy making. If these disenfranchised/disadvantaged populations are prioritized and simply placed high on a list, they are likely to be vaccinated at relatively low rates, as we would expect with disenfranchised and marginalized populations. This will continue to place society at risk^12^. In other words, in this short paper we do not only want to emphasize the need for moral equality -which is commendable- but we also want to point out that honoring moral equality also guarantees the society’s efficient functioning and is to the benefit of the whole community, as brilliantly noted by bioethicist *Mark Kuczewski* in two recent pieces^13^ ^,^ ^14^.

The need to consider the special needs of these three groups with respect to vaccine allocation also emerges in light of the proposal to introduce a digital corona passport. Denmark was among the first countries to promote the adoption and implementation of such a passport, quickly followed by many European states. The digital corona passport is an app that should, in the mind of the developers, enable people to prove they were vaccinated against Covid-19 or otherwise immunized^15^. Those who could prove they are protected from the virus will be able to return to international travel, to eat in restaurants, to go to the cinema or to the theater, or even to participate in mass events (such as concert), as before the pandemic, while those who haven’t been vaccinated couldn’t.

The combination of vaccination, a corona passport attesting to immunization, and the opening of activities reserved for those who are in possession of the necessary electronic document seems nevertheless to be opening the way for the establishment of even stronger inequalities between citizens, which may not be justified, even if they are considered to be temporary.

In this context, though, we should not forget that SARS-CoV-2 is a virus capable of rapid mutations and that Covid-19 could become an endemic disease. In this scenario, it may become necessary to repeat vaccinations every year and it cannot be excluded that new variants of the virus will require periodic modifications of our vaccines. All this could mean that, at least in some stages, vaccine shortages would not be a rare phenomenon and that -as a consequence-stronger inequalities could turn out to be a particularly pervasive phenomenon of our future societies. All that considered, we believe that we should now stand for both equality and equity more loudly and seek the development of more inclusive public health policies as priorities, at least if we wish to call our society a just society.

As we have seen above, some disadvantaged groups risk not being sufficiently valued in vaccine distribution plans. This may put their health and the health of society at risk, while contributing to betraying the ideals (of fairness and equality) to which many of us would subscribe and are indeed committed. We therefore feel we ought to introduce, as we have suggested above, some corrective measures in vaccination plans in order to make them more equitable and more effective. More importantly, we believe that such plans (for COVID-19 as well as for other future diseases) should have more targeted and inclusive outreach and be implemented as systemic efforts capable of adequately and effectively protecting marginalised populations and disadvantaged/disenfranchised groups [41–44]. This will contribute to avoid present and perhaps future discriminations, which may also have undesirable consequences at several levels (ethical, political etc).

For this reason, we should ensure that the criteria with which vaccines are allocated are the most efficient from a medical and an epidemiological standpoint. However, such criteria should also be formulated, so as to envisage priority inclusion of disadvantaged categories [41, 42]. Moreover, we believe that these criteria should be inspired by principles of equity, equality, inclusiveness, and fairness, which should all be safeguarded in order to keep the society cohesive and capable of distributing costs and benefits according to justice.

However, one cannot forget about the economic as well as the political costs related to this type of health care choice. Obviously, inclusion and prioritization do have costs. A country, a region, or a city council need to hire specialized health personnel to reach out and vaccinate marginalised populations and disadvantaged/disenfranchised groups. These costs can be very high, especially if compared to the costs of a simple injection at a mass vaccination centre. Nevertheless, it is important to note -as indicated above-that the protection of all vulnerable segments of society helps the entire community to contain the pandemic, -for instance-by preventing the emergence of new dangerous variants. This form of protection ultimately translates into direct and indirect economic savings that offset the costs for initial vaccinations.

There may be a political cost involved in this type of health care choice as well. Minorities, marginalized populations, and disadvantaged groups typically have little voice in the public arena, and citizens -who generally support the administrations in charge-may not favour the inclusive health policies we have just described. This may happen because of the myopia with which they look at the issue, considering only the short-term costs and not assessing the potential medium-long term gains (we are not counting here the intrinsic value of the moral action underlying such interventions, which obviously deserve praise in itself).

Surely the potential costs (both economic and political) of inclusion and prioritization of the most disadvantaged groups could constitute an obstacle to the implementation of the health policies that we have defended in this paper. The analysis we have conducted and the reasons we have proposed could, however, help convincing governments and policymakers to make more ethical and far-sighted choices, in the face of possible short-term criticisms coming from their constituencies.In our view the adoption of more inclusive vaccine policies goes in the direction of an efficient society that lives up to its best ideals when considering the fundamental domain of citizens’ health.

## Footnotes

1
https://www.who.int/publications/m/item/draft-landscape-of-covid-19-candidate-vaccines Last accessed August 2021

2
https://www.businesswire.com/news/home/20210205005496/en/Sinovac-Announces-Phase-III-Results-of-Its-COVID-19-Vaccine Last accessed August 2021

3
https://www.cdc.gov/coronavirus/2019-ncov/vaccines/recommendations.html Last accessed August 2021

4
https://www.gov.uk/government/publications/priority-groups-for-coronavirus-covid-19-vaccination-advice-from-the-jcvi-30-december-2020/joint-committee-on-vaccination-and-immunisation-advice-on-priority-groups-for-covid-19-vaccination-30-december-2020#vaccine-priority-groups-advice-on-30-december-2020 Last accessed August 2021

5 Interestingly [18], take a different perspective on this. They argued that in order to maximize the benefits of indirect immunity for the elderly and the other vulnerable groups we ought to first immunize adolescents and the youngest sectors of society

6
https://www.who.int/immunization/sage/meetings/2020/october/Session03_Roadmap_Prioritization_Covid-19_vaccine.pdf Last accessed August 2021

7
https://www.statnews.com/2021/03/11/the-uncounted-people-who-are-homeless-are-invisible-victims-of-covid-19/Last accessed August 2021. Thanks to the reviewer for pressing us on this point

8
https://www.theguardian.com/society/2020/may/29/san-francisco-homeless-deaths-coronavirus Last accessed August 2021

9
https://capitalandmain.com/homeless-deaths-in-los-angeles-rose-by-more-than-30-percent-in-2020-0202 Last accessed August 2021

10
https://www.washingtonpost.com/gdpr-consent/?next_url=https%3a%2f%2fwww.washingtonpost.com%2flocal%2fdc-homeless-deaths%2f2020%2f12%2f30%2fe94d74fc-453e-11eb-8deb-b948d0931c16_story.html Last accessed August 2021

11
https://www.reuters.com/article/uk-health-coronavirus-britain-immigrants/all-migrants-living-in-uk-eligible-for-covid-19-vaccine-idUSKBN2A80ZR Last accessed August 2021

12 Thanks to the reviewer for pressing us on this point

13
http://www.bioethics.net/2021/01/deny-vaccination-to-undocumented-immigrants-who-might-benefit/Last accessed August 2021

14
https://thehill.com/opinion/immigration/451937-health-insurance-for-undocumented-immigrants-its-only-fair-to-all-of-us Last accessed August 2021

15
https://www.technologyreview.com/2021/02/10/1017880/denmark-coronavirus-passport-covid-immunity/ Last accessed August 2021
